# Genetic diversity and population structure of the human malaria parasite *Plasmodium falciparum* surface protein Pfs47 in isolates from the lowlands in Western Kenya

**DOI:** 10.1371/journal.pone.0260434

**Published:** 2021-11-29

**Authors:** Shirley A. Onyango, Kevin O. Ochwedo, Maxwell G. Machani, Collince J. Omondi, Isaiah Debrah, Sidney O. Ogolla, Ming-Chieh Lee, Guofa Zhou, Elizabeth Kokwaro, James W. Kazura, Yaw A. Afrane, Andrew K. Githeko, Daibin Zhong, Guiyun Yan

**Affiliations:** 1 Department of Zoological Sciences, School of Science and Technology, Kenyatta University, Nairobi, Kenya; 2 Sub-Saharan Africa International Centre of Excellence for Malaria Research, Homa Bay, Kenya; 3 Department of Biology, Faculty of Science and Technology, University of Nairobi, Nairobi, Kenya; 4 Centre for Global Health Research, Kenya Medical Research Institute, Kisumu, Kenya; 5 Department of Biochemistry, Cell and Molecular Biology, West African Centre for Cell Biology of Infectious Pathogens, University of Ghana, Accra, Ghana; 6 Program in Public Health, College of Health Sciences, University of California, Irvine, California, United States of America; 7 Center for Global Health and Diseases, Case Western Reserve University, Cleveland, Ohio, United States of America; 8 Department of Medical Microbiology, University of Ghana Medical School, University of Ghana, Accra, Ghana; Ehime Daigaku, JAPAN

## Abstract

*Plasmodium falciparum* parasites have evolved genetic adaptations to overcome immune responses mounted by diverse *Anopheles* vectors hindering malaria control efforts. *Plasmodium falciparum* surface protein Pfs47 is critical in the parasite’s survival by manipulating the vector’s immune system hence a promising target for blocking transmission in the mosquito. This study aimed to examine the genetic diversity, haplotype distribution, and population structure of Pfs47 and its implications on malaria infections in endemic lowlands in Western Kenya. Cross-sectional mass blood screening was conducted in malaria endemic regions in the lowlands of Western Kenya: Homa Bay, Kombewa, and Chulaimbo. Dried blood spots and slide smears were simultaneously collected in 2018 and 2019. DNA was extracted using Chelex method from microscopic *Plasmodium falciparum* positive samples and used to genotype Pfs47 using polymerase chain reaction (PCR) and DNA sequencing. Thirteen observed haplotypes of the Pfs47 gene were circulating in Western Kenya. Population-wise, haplotype diversity ranged from 0.69 to 0.77 and the nucleotide diversity 0.10 to 0.12 across all sites. All the study sites displayed negative Tajima’s *D* values although not significant. However, the negative and significant Fu’s *Fs* statistical values were observed across all the study sites, suggesting population expansion or positive selection. Overall genetic differentiation index was not significant (F_*ST*_ = -0.00891, P > 0.05) among parasite populations. All Nm values revealed a considerable gene flow in these populations. These results could have important implications for the persistence of high levels of malaria transmission and should be considered when designing potential targeted control interventions.

## Introduction

*Plasmodium falciparum* is accountable for a majority of malaria infections and deaths in the African region accounting for 99.7% of estimated malaria cases in 2018 [1]. The endemic Lake regions (risk class equal to or above 20%) majorly lowlands have most of the malaria burden in Kenya and transmission occurs throughout the year [2, 3]. The combination of control interventions used in these regions includes long-lasting insecticidal nets, indoor residual spray and Artemisinin-based combination treatments [4]. Yet, there is still continuous transmission of the *P*. *falciparum* among the vulnerable populations. The spread of malaria is a result of the parasites’ adaptation to indigenous vectors in different geographical regions [5]. Hence, over 70 *Anopheles* species can transmit *P*. *falciparum* malaria [6]. The mosquito immune system can significantly deter successful malaria transmission and is critical for controlling the vector capacity [7]. For a complete transmission circuit, *Plasmodium* parasites have to overcome immune responses mounted by diverse *Anopheles* vectors [8–10]. Pfs47 is a surface protein in *P*. *falciparum* expressed on the surface of female gametocytes, gametes, zygotes and ookinetes [11] that interacts with the mosquito midgut making the parasite invisible to the vector’s immune system thus providing the parasite with an immune evasion mechanism [12] limiting the efforts to effectively control and eliminate malaria. Hence, Pfs47 is a potential molecular target of interest in designing appropriate interventions for malaria [12–14].

The Pfs47 gene is exceptionally polymorphic with a strong geographical genetic structure and diversity [15, 16]. It exhibits haplotypes that are naturally selected by the anopheline vectors in varying geographical regions causing significant variations in malaria transmission [13, 16]. *Plasmodium falciparum* isolates from African strains have consistently displayed high levels of genetic diversity [14, 17, 18] and a strong geographic structure in the Pfs47 gene from laboratory and field isolates [16, 19] as well as haplotypes found circulating within the major malaria vectors; *An*. *gambiae* and *An*. *funestus* populations. These results were a clear indication that compatible Pfs47 haplotypes are naturally selected within vector populations in Africa triggered by the mosquito’s immune pressures [16]. Previous findings identified 42 Pfs47 haplotypes that exhibit high dN/dS worldwide [13] and the evolutionary relationships between these haplotypes revealed 32 haplotypes exclusively from Africa, Papua New Guinea, the Americas, and Asia [20]. These polymorphisms may therefore have a significant impact on the trends in malaria transmission dynamics and the parasite history.

*Plasmodium falciparum* genetic diversity and population structure determined by various factors including transmission intensity and levels of inbreeding in varied endemicities [21, 22], human and vector movement [23], geographical features that create barriers or promote gene flow [21], and locally implemented malaria control interventions [19, 24] are critical in designing targeted malaria control measures. However, knowledge on the underlying mechanisms that these *Plasmodium* parasites adapt to major malaria vectors from differing regions and endemicities is limited. The need to conduct refined local *Plasmodium* parasites population genetics that will significantly improve our understanding of transmission dynamics and contribute to designing targeted control and management tools against malaria is vital. This study evaluated the genetic diversity, haplotype distribution, and population structure of Pfs47 and its implications on malaria infections in endemic regions in Western Kenya.

## Materials and methods

### Study sites and sampling

**Fig 1:**
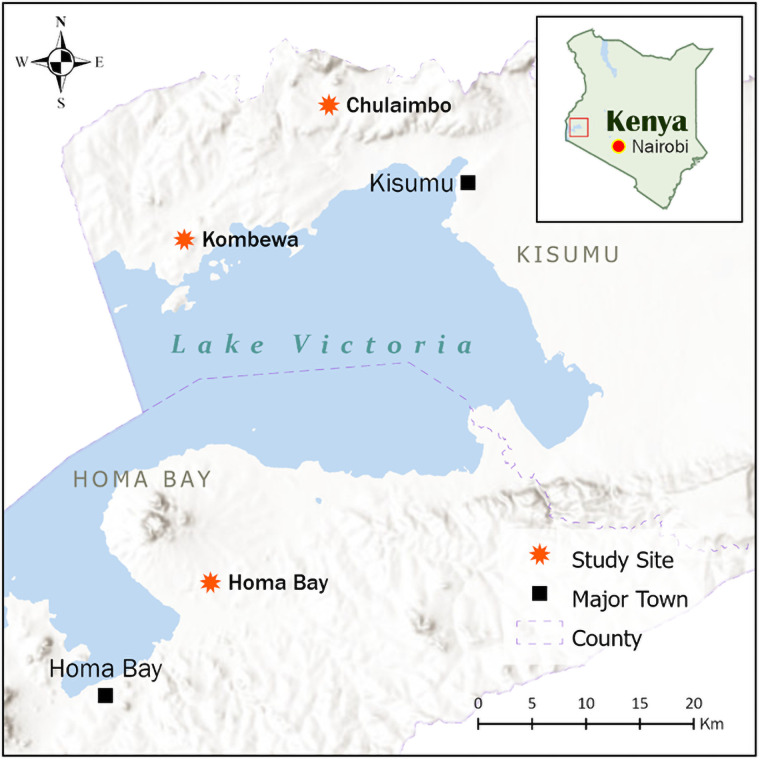
Map of Western Kenya showing the sampling locations. The map was generated using ArcGIS Pro 2.6 software. Map source: ESRI, CGIAR, and USGS (available at: www.esri.com).

Cross-sectional blood screening was conducted in January to August 2018 and January to March 2019 in three malaria-endemic regions in the lowlands of Western Kenya: Kombewa (34°30′E, 00°07′N; altitude ranges 1,170–1,300 m above sea level), Chulaimbo, a rural site 19 km west of Kisumu City (0.03572°S, 34.621°E, altitude ranges 1328–1381 m above sea level) and Homa Bay (0.3800 S, 34, 6419 E, altitude 1300 m) (Fig 1). Malaria transmission is perennial in the lowland and the major *Plasmodium* transmitters in these regions include *An*. *gambiae* and *An*. *arabiensis* [25]. Malaria incidence in the lowlands is consistently high and is characterized by flat land with vast malaria breeding habitats especially during the rainy season [26]. Kombewa is semi-arid with poor drainage and semi-permanent swampy streams and an average monthly temperature range of 18.4°C–29.1°C [27]. Malaria is holoendemic in this region and transmission occurs throughout the year. The economic activities in Kombewa involve subsistence farming, animal husbandry, and fishing [28]. Chulaimbo has a sporadic water supply system, and limited sewer and waste disposal, and a mean annual temperature range of 12°C–35°C. The region experiences an average annual rainfall of 1352 mm and an average relative humidity range of 66–83%. Most residents are small-scale subsistence farmers. Homa Bay has extensive environmental modifications and human migration and experiences semi-arid climatic conditions and depends on the Kimira-Oluch irrigation scheme for food production.

### Parasitological surveys

Blood samples were collected from participants of different ages (<5, 5–15, >15 years) who had consented to the study. Dried blood spots (DBS) were collected by finger prick on well-labeled Whatman^®^ 903 Protein Saver Cards (GE Healthcare WB100014) containing the participants’ details. Approximately, 50 μl of blood was drawn and placed onto the Whatman^®^ 903 Protein Saver Cards and allowed to air dry before they were individually stored and preserved at -20°C for molecular analyses. Thick and thin smears were simultaneously prepared for *Plasmodium* species identification and parasite counts. Blood films were stained using 10% GIEMSA and examined in a compound microscope to determine the presence of *Plasmodium* parasites. Only the 125 DBS from *P*. *falciparum* positive participants were used to genotype Pfs47 and later sequenced to determine haplotypes distribution.

### DNA extraction and *Plasmodium* species identification

*Plasmodium falciparum* parasite DNA was extracted from the DBS using Chelex method [29]. Briefly, 3 mm discs were cut from each of the DBS were soaked in 10% Saponin and Phosphate buffer saline (PBS), and incubated overnight. The preparation was washed twice in 1xPBS and boiled in 20% (wt/vol) chelex suspension (styrene-divinylbenzene co-polymer containing iminodiacetic acid groups). The mixture was vortexed and centrifuged and used to identify *Plasmodium* species as described elsewhere [30]. In brief, multiplex real-time PCR (RT-PCR) was run in a final volume of 12 μl containing 2 μl of sample DNA, 6 μl of PerfeCTa^®^ qPCR ToughMix^™^, Low ROX^™^ Master mix (2X), 0.5 μl of each species specific probe including *P*. *falciparum*, *P*. *ovale* and *P*. *malariae* 0.4 μl of each species specific forward primers (10 μM), 0.4 μl of each species specific reverse primers (10 μM) and 0.1 μl of double-distilled water. (Primers and probes sequences are shown in S1 Table). The thermal profile used was 50 °C for 2 min, (95 °C for 2 min, 95 °C for 3 sec and 58 °C for 30 sec) for 45 cycles. After species confirmation by RT-PCR, 125 samples that were infected with *P*. *falciparum* parasites only were randomly selected for genotyping the Pfs47 gene.

### Genotyping Pfs47

Genotyping Pfs47 was performed following the method as described by Anthony et al. [16] with modifications. Briefly, forward 5’ATGTGTATGGGAAGAATGATCAG3’ and reverse 5’ACAAGTTCATTCATATGCTAACATA3’ primers were used to amplify the entire coding region 1320 bp from the DNA of the 125 *P*. *falciparum* positive samples. A final reaction volume of 12 μl was prepared by addition of 6 μl of Dream Taq Green PCR Master Mix (2X), 0.5 μl of each of the forward and reverse primer, 3 μl of double distilled PCR grade water, and 2 μl of sample DNA. The PCR conditions were set as follows; 95°C for 3min, 35X (94°C for30 sec, 50°C for 30 sec, 68°C for 90 sec), and 72°C for 6 minutes before sequence, amplicons quality and size were determined by visualization of PCR products in 1.5% w/v gel under UV transilluminator. The amplicons were cleaned and sequenced directly using BigDye terminator chemistry v3.1, PCR primers, and PRISM^®^ 3730xl genetic analyzer (Applied Biosystems, CA, USA). Paired reads from the sequencer were edited and assembled using BioEdit software (version 7.2.5) before further analysis.

### Ethics approval

The study was approved by the Maseno University Ethics Review Committee (MUERC protocol No. 00456) and the University of California, Irvine Institutional Review Board (UCI IRB) and received authorization from the Ministry of Health, Kenya. All volunteers or their guardians gave written informed consent to participate in providing blood samples for the study.

### Data analysis

The 125 assembled sequences were aligned with reference to Pf3D7_1346800 using ClustalW algorithm (in-built in Mega X software) and DnaSP Version 6.12.03 was used to compute genetic diversity indices such as nucleotide diversity, mean pairwise differences, polymorphic sites, haplotype diversity, and linkage disequilibrium. Population Analysis with Reticulate Trees (Popart) version 1.7 software was used to construct haplotypes network showing the distribution of haplotypes per study site. MEGA software was used to construct the UPGMA (unweighted pair group method with arithmetic mean) tree based on the Kimura 2-parameter (K2P) distance model with 1,000 bootstrap replicates. Allelic, genotypic frequency, and population genetics (fixation index, gene flow, and Analysis of molecular variance) were inferred using GenAlEx version 6.5 software. The analysis of molecular variance (AMOVA) was categorized into among populations/groups representing the three lowland sites *P*. *falciparum* populations (Kombewa, Chulaimbo, and Homa Bay), among populations within populations and individuals within groups.

## Results

A total of 1518 participants were screened for malaria parasites from the three study sites. Out of 1518, 20.5% (309/1518) were positive for *P*. *falciparum*. The *P*. *falciparum* prevalence was 25.5%, 8.9%, and 56.8% from Chulaimbo, Homa Bay, and Kombewa respectively. One hundred and twenty five samples (67 were female and 58 males) were randomly selected for Pfs47 gene sequencing and analyses.

### Genetic diversity indices of Pfs47 across Western Kenya

*Plasmodium falciparum* parasites (n = 125) from Homa Bay (n = 62), Chulaimbo (n = 30), and Kombewa (n = 33) were successfully sequenced from Western Kenya lowlands (Fig 1). Nucleotide sequence analysis of the Pfs47 gene compared to Pf3D7_1346800 revealed 8 segregating sites, (6 parsimony informative sites, and 2 singletons or SNPs). Single nucleotide polymorphisms (SNPs) were observed at mutation loci 581 and 814 whereas 81, 564, 718, 742, 815, and 910 were parsimony informative. Generally, Pfs47 from parasites populations in Homa Bay and Kombewa displayed relatively high genetic diversity as compared to the Chulaimbo region (Table 1). The distribution and relative frequencies by population have been shown in Table 2. Overall haplotype diversity (*Hd*) and nucleotide diversity (*π*) values were 0.74±0.03 and 0.11±0.01, respectively. Population-wise, haplotype diversity values ranged from 0.69 to 0.77 and 0.10±0.02 to 0.12±0.01 for nucleotide diversity which was generally low across all sites (Table 1).

**Table 1:** Genetic diversity indices and neutrality tests based on Pfs47 sequences.

Population	n	*S*	*H*	*H*d	*π* (10^−2^)	*k*	Fu’s *Fs*	Tajima’s *D*	Fu and Li’s *D*	Fu and Li’s *F*
Homa Bay	62	7	11	0.77±0.04	0.12±0.01	1.22	-4.95*	-0.46	-0.39*	-0.49*
Kombewa	33	7	10	0.76±0.07	0.11±0.02	1.21	-5.47*	-0.87	-0.16*	-0.44*
Chulaimbo	30	5	7	0.69±0.08	0.10±0.02	1.05	-2.58*	-0.47	-0.61*	-0.66*
Overall in Western Kenya	125	44	13	0.74±0.03	0.11±0.01	1.17	-5.90*	-0.49	-0.42*	-0.53*

n, number of samples sequenced; *S*, number of polymorphic (segregating) sites; *H*, number of Haplotypes; *H*d, Haplotype diversity; *π*, nucleotide diversity; *k*, mean number of pairwise differences.

* indicates the significance P<0.05.

**Table 2:** Pfs47 allele frequencies per mutation loci.

Mutated loci	Pfs47 Domains	Genotypic frequencies % (n)		
		Homa Bay (n = 62)	Chulaimbo (n = 30)	Kombewa (n = 33)
**81**	D1	61.29 (38)	70 (21)	66.67 (22)
**564**	D2	6.45 (4)	13.33 (4)	9.09 (3)
**581**	D2	1.61 (1)	0	0
**718**	D2	20.97 (13)	13.33 (4)	9.09 (3)
**742**	D2	6.45 (4)	3.33 (1)	9.09 (3)
**814**	D3	0	0	3.03 (1)
**815**	D3	1.61 (1)	3.33 (1)	6.06 (2)
**910**	D3	4.84 (3)	0	3.03 (1)

n is the total number tested.

Dimorphic locus 81 was the most predominant with >0.5 allele frequency across Pfs47 sequences from the three sites (Table 2). In Homa Bay, 61.29% of sequences had mutations at locus 81 while 6.45%, 1.61%, 20.97%, 6.45%, 1.61% and 4.84% sequences had mutations at loci 564, 581, 718, 742, 815 and 910 respectively. None of the sequences had a mutation at locus 814. Sequences from Chulaimbo lacked mutations at locus 581, 814, and 910 but had mutations at loci 81, 564, 718, 742, 815 at frequencies of 70%, 13.33%, 13.33%, 3.33%, and 3.33% respectively. The *P*. *falciparum* populations from Kombewa lacked mutations at locus 581 but had mutations at loci 81, 564, 718, 742, 814, 815, and 910 at frequencies of 66.67%, 9.09%, 9.09%, 9.09%, 3.03%, 6.06% and 3.03% respectively.

All the observed base substitutions on the 8 loci resulted in 8 nonsynonymous changes E27D, E188D, P194H, L240I, I248L, N272I, N272Y, and I304L on the Pfs47 amino acid chain. Homa Bay and Kombewa populations had the highest number of segregating sites, unlike the Chulaimbo parasite populations. Homa Bay parasites displayed a slightly higher nucleotide diversity of 0.12±0.01 compared to Kombewa and Chulaimbo parasites (Table 1). Sequences from all study sites deviated from the standard neutral and displayed negative Tajima’s *D* values upon subjection to the neutrality test. The Tajima’s *D* values were however not significant thus displaying a weak selection within and among all study sites. Other neutrality tests result for each study site Homa Bay Fu and Li’s *D* -0.39 and Fu and Li’s *F* test -0.49, Kombewa Fu and Li’s *D* -0.16 and Fu and Li’s *F* -0.44, Chulaimbo Fu and Li’s *D* -0.61 and Fu and Li’s *F* -0.66, all sites Fu and Li’s *D* -0.42 and Fu and Li’s *F* -0.53 were significant (Table 1). The Fu’s *Fs* statistics were however significant across all the study sites indicating the presence of excess frequency of rare haplotypes across sites in Western Kenya.

**Fig 2:**
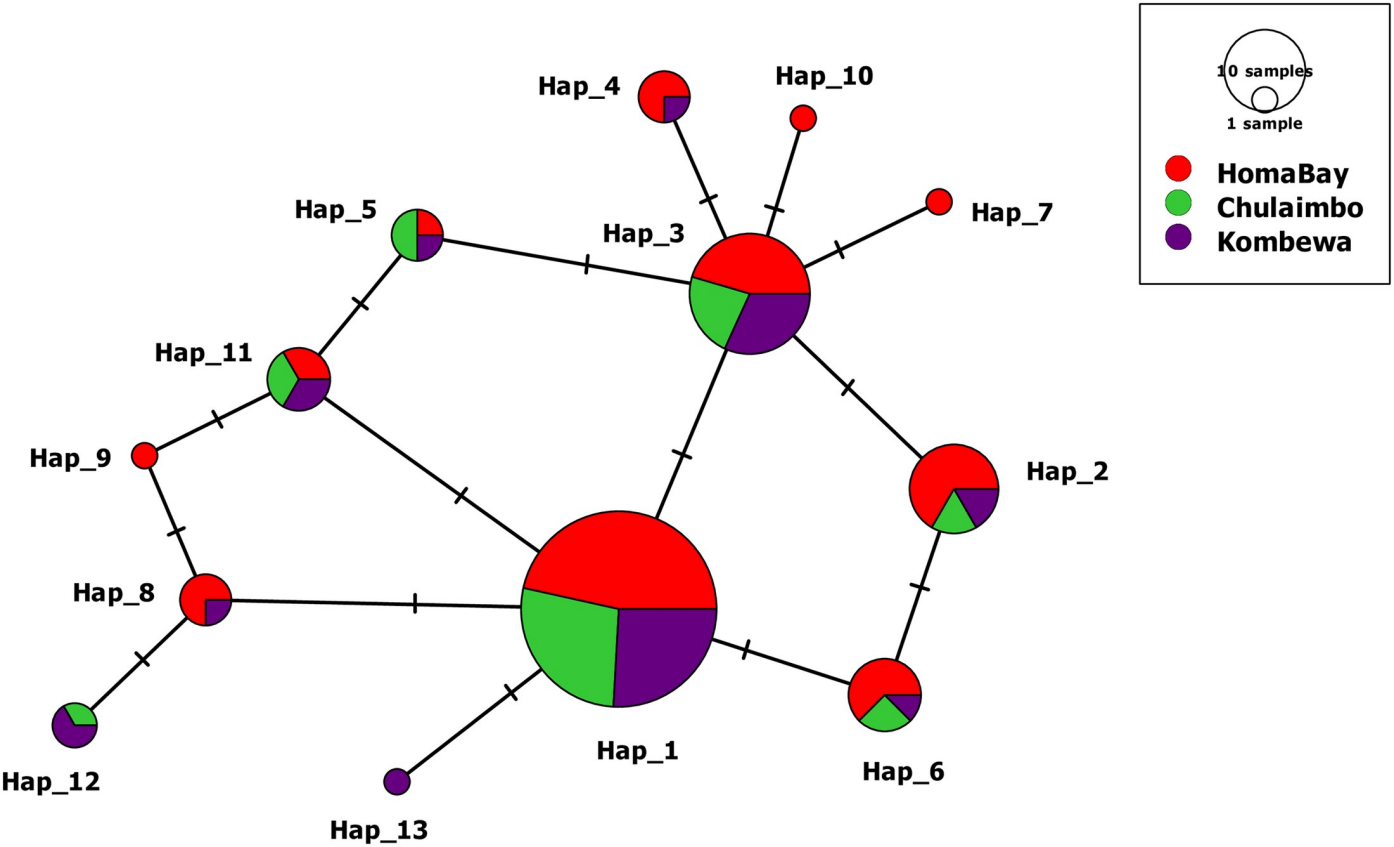
TCS-network of Pfs47 haplotypes showing all variants identified in Western Kenya regions (Hap_1-Hap_13). The size of the circle corresponds to haplotype frequency and the hatch mark represents the number of mutations observed. Colors represent circulating haplotypes identified in Homa Bay, Chulaimbo, and Kombewa.

Overall, 13 different haplotypes were identified across sampled sites. A total of 11 haplotypes were identified circulating within *P*. *falciparum* parasites in Homa Bay whereas 10 and 7 were observed in the Kombewa and Chulaimbo populations respectively (Table 1). The TSC network shows haplotype distribution among the three regions (Fig 2; S2 Table). The haplotype distribution shows that Hap_1, 2, 3, 5, 6, and 11 are shared across all populations. Hap_3 is widely spread and is likely to be the ancestral variant. A slightly high *H*d (0.77±0.04) was observed in Homa Bay. Hap_7, 9, and 10 were exclusively identified in the Homa Bay populations whereas Hap_13 was only observed in Kombewa. All other haplotypes were found circulating in respective study sites at different proportions. TCS network profile of 13 haplotypes indicated that all haplotypes were connected by one mutation step between haplotypes (Fig 2). S2 Table shows the mutations between the haplotypes identified in this study.

### Phylogenetic relationship of Pfs47 haplotypes

**Fig 3:**
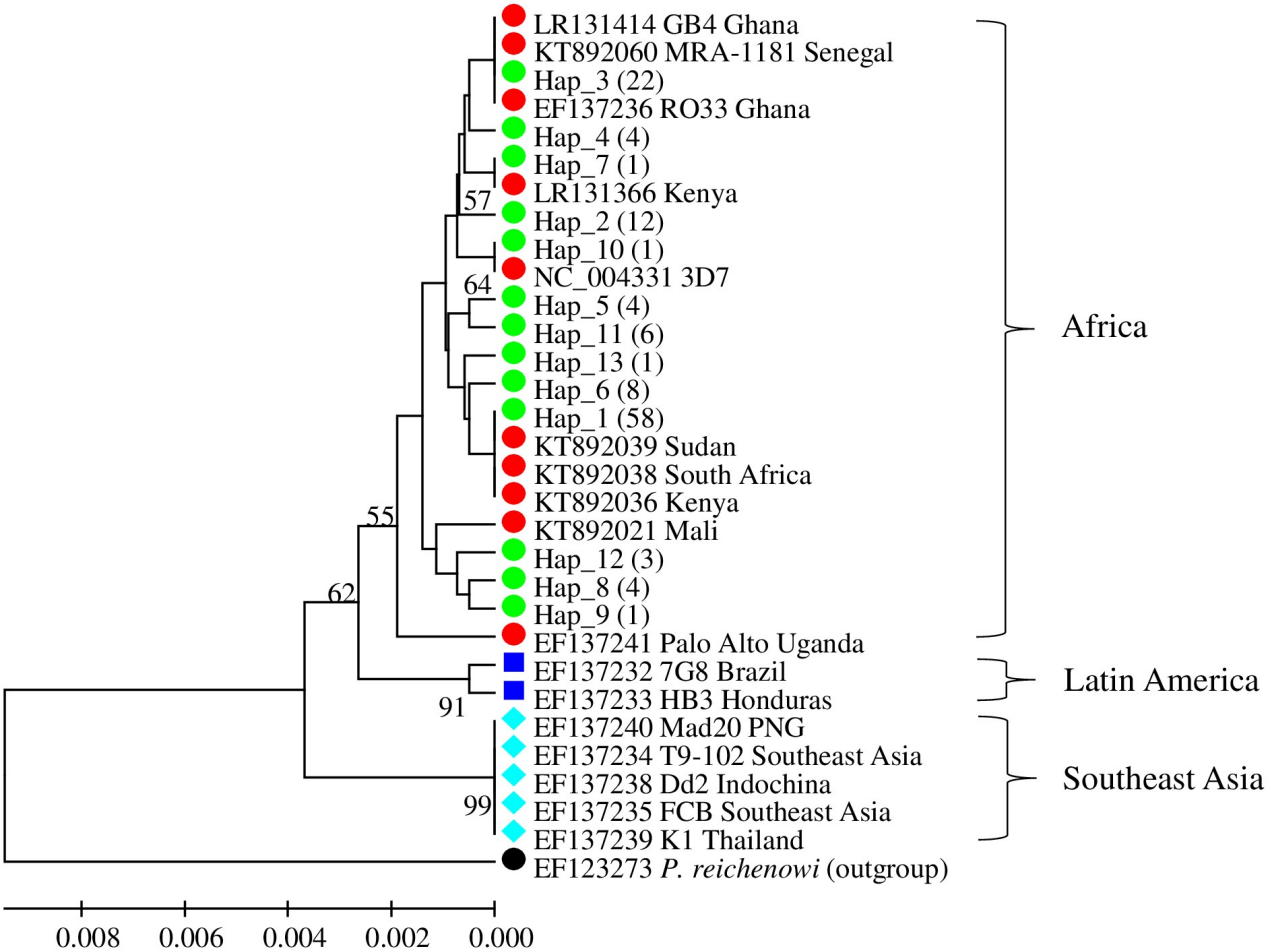
Phylogenetic inference using UPGMA method. The percentage of replicate trees (>50) in which the associated pfs47 haplotypes clustered together in the bootstrap test (1000 replicates) are shown next to the branches. The evolutionary distances were computed using the Kimura 2-parameter method and are in the units of the number of base substitutions per site. The tree was rooted to a reference sequence from *P*. *reichenowi* and the scale axis presented under the tree. Green dots represent the haplotypes identified in this study in Western Kenya, values in parentheses indicate the number of sequences, red dots represent haplotypes previously identified from other studies in African countries, blue filled squares represent haplotypes from Latin America, the aqua diamond represents haplotypes from Southeast Asia, and the black dot represents the outgroup. Eighteen sequences were retrieved from GenBank: (NC_004331, EF137232, EF137233, EF137234, EF137235, EF137236, EF137238, EF137239, EF137240, EF137241, LR131414, KT892038, KT892060, KT892036, KT892039, LR131366, KT892021, EF123273).

A UPGMA phylogenetic tree of the Pfs7 haplotypes was generated from 1,000 bootstrap replicates of the K2P distance matrices (Fig 3). Phylogenetic analysis showed that all *P*. *falciparum* haplotypes clustered into three major haplogroups (Africa, Latin America, and Southeast Asia) with moderate to high bootstrap support values, ranging from 55% to 99%, suggesting strong geographic structure in natural *P*. *falciparum* populations from different continents. Out of the sequences retrieved from the gene bank, Pfs47 haplotypes from western Kenya have a common ancestral lineage with haplotypes from other African countries. Hap_1, the most common haplotype is clustered with haplotypes from East Africa (Sudan and Kenya) and South Africa, whereas Hap_3, the second most common haplotype is grouped with those from West Africa (Ghana and Senegal). Hap_7 and Hap_10 each were identified in one sample and clustered with LR137236 (Kenya) and NC_004331 (3D7), respectively. Overall, there was poor bootstrap support (<50%) for the grouping of the rest haplotypes.

### *Plasmodium falciparum* population structure and differentiation based on Pfs47 sequences

There was no significant difference in *P*. *falciparum* parasite populations circulating in the three regions. All the inter-population comparison chi-squares values (Kombewa vs Chulaimbo *x*^2^ = 4.232, df = 1, P = 0.895, Kombewa vs Homa Bay *x*^2^ = 10.91, *df* = 1, P = 0.618, Homa Bay vs Chulaimbo *x*^2^ = 10.39, *df* = 1, P = 0.582) were not significant (P>0.05) (Table 3). All Nm values were more than two suggestive of a considerable gene flow in these populations (Table 3). Kombewa and Chulaimbo had similar GammaSt Nm values when the populations were each compared to Homa Bay. The two study sites, Chulaimbo and Kombewa are within proximity and approximately equally far from Homa Bay study sites. Pairwise *F*_ST_ values between populations were zero suggesting that there was no genetic differentiation among the populations (Table 3).

**Table 3:** Population structure and gene flow among *Plasmodium falciparum* across Western Kenya regions.

Populations		χ ^2^	P-value	*df*	*F*_ST_	GammaSt Nm
Kombewa	**Chulaimbo**	4.232	0.895	9	-0.023	51.000
Kombewa	**Homa Bay**	10.911	0.618	13	-0.004	34.200
Homa Bay	**Chulaimbo**	10.394	0.5815	12	-0.003	34.620

χ ^2^ Chi-square; *df*, degrees of freedom; *F*_ST_, fixation index_;_ Nm, gene flow estimate.

The analysis of molecular variance (AMOVA) results indicate that 100% of the observed variations in allele frequency were among individuals within respective populations, and no variation (0%) was observed among populations and within individuals (Table 4). Wright’s F-statistic indicated an insignificant population structure and the overall genetic differentiation index (*F*_*ST*_) was -0.00891 (P>0.05) among parasite populations from all the sites.

**Table 4:** Analysis of molecular variance of Pfs47 gene in *P*. *falciparum* population circulating in Homa Bay, Chulaimbo and Kombewa.

Global AMOVA results					
Source of variation	*df*	Sum of squares	Mean Square	Established Variation	Percentage of variation
Among populations	2	1.681	0.841	0.000	0
Among Individual	122	143.439	1.176	0.588	100
Within Individuals	125	0.000	0.000	0.000	0
Total	249	145.120		0.588	100

*df*, degrees of freedom.

## Discussion

The genetic diversity of *P*. *falciparum* immune selected antigens is critical in the parasite’s ability to circumvent or evade its host immune system [8]. The selection pressure from Anopheles mosquitoes is hypothesized to shape the distribution of Pfs47 haplotypes in regions with varying transmission intensities. Pfs47 displayed a high haplotype diversity with a varying number of haplotypes circulating within the human population per studied region. Due to the lack of geographical barriers among the three regions with varying transmission intensities, there were high levels of gene flow and low parasite population structure. The neutrality test results revealed that the Pfs47 gene may be under purifying selection pressures suggestive of a recent population expansion in malaria endemic areas.

*Plasmodium falciparum* is diverse and has varying patterns of population genetic characteristics that correlate with local endemicities and transmission intensity [21]. Reports have shown that the population genetic diversity of *P*. *falciparum* tends to be low in hypo to meso-malaria endemic regions and high in hyperendemic regions [21, 31]. These results also demonstrate similar trends where parasites from Chulaimbo a meso-endemic region had the lowest genetic diversity compared to the Homa Bay and Kombewa *P*. *falciparum* populations. In this study, the Pfs47 gene has displayed relatively diverse haplotypes with low nucleotide diversity being observed across sites within Western Kenya. Homa Bay (hyperendemic) had the highest nucleotide diversity followed by Kombewa (holoendemic) then Chulaimbo (mesoendemic). The high diversity corresponds to the observed mean pairwise differences and haplotype diversity per study site. The variation in genetic diversity indices of Pfs47 per site corroborates previous results that linked various parasite genetics to levels of malaria transmission intensities [32, 33]. Most (50%) of the observed mutations occurred within immunogenic domain two (D2) of Pfs47 antigen which is in agreement with findings from previous studies [13]. The variations within D2 of Pfs47 antigen has been hypothesized to be vital and aid parasite in escaping nitration or TEP1 mediated killing [34]. Domain 3 had only three mutations while domain 1 had one which was much pronounced or had high allele frequencies across the three study regions. All the sequences from each site except one from Homa Bay had at least one of the loci mutations. The sequence lacking mutation corresponds to NF54 wild strains that were reported to have over 90% chance of survival in the *An*. *gambiae* R strain mosquitoes [13, 34].

Out of the 13 observed Pfs47 haplotypes, 6 haplotypes were shared in the three regions representing the different transmission intensities. However, haplotypes harboring mutation codon E27D were predominant in each site and seem to be highly selected or most infective within the Western Kenya *P*. *falciparum* populations. This finding reaffirms parasites having mutation codon E27D to be the most predominant Pfs47 haplotype only found in *P*. *falciparum* parasites circulating in Africa [13]. Consistent with findings from another study [13], other common haplotypes most of which had mutations in D2 and were found in parasites from the three study regions were E188D, L240I, I248L, and N272Y. Among the four mutation codons, I248L is more conservative and results in a change of methyl group position within the side chain also identified by Canepa et al. [34] and Eldering et al. [35] in *P*. *falciparum* African strains. The mutations are shown to slightly increase infection rates to 4% non-silenced and further 75% in *A*. *albimanus* with silenced LRIM1 [34]. Apart from mutation codon I304L described here for the first time, the other seven have been described in parasites circulating in Africa, Asia, America, and Papua New Guinea [13]. Haplotypes with mutation codon I304L were unique to the Kombewa and Homa Bay parasite populations whereas those with mutation codon P194H and N272I were private to Homa Bay and Kombewa populations respectively. Compared to the Pfs47 orthologue Pvs47 (PVX_083240), both share a 38.5%-38.7% amino acid identity, and the haplotype distribution exhibit a geographical population structure indicative of alleles favored by natural selection in a given region [36]. Not all the observed mutations in Pfs47 were present in Pvs47, however, in amino acid sequences Pfs47 from Western Kenya had two mutation sites (27 and 240) at same loci position as the one described in Pvs47 [36].

All the inter-population comparisons displayed non-significant differences across the three *P*. *falciparum* populations thus confirming a weak population structure. The weak population structure or lack of significant difference in nucleotide diversity indices may be as a result of considerable gene flow, lack of geographical barriers, and inbreeding characterizing parasites at various sites in Western Kenya. Furthermore, Western Kenya has a vast network of roads that facilitate movement and trade across these study regions. Human movement may also affect the parasite population structure by introducing an admixture of *P*. *falciparum* strains [37, 38] as a result of a weak structure as illustrated in this study. *Plasmodium falciparum* populations from Homa Bay and Kombewa showed the strongest evidence of endemic structure. This is consistent with other studies conducted in the African continent where *P*. *falciparum* is diverse and has varying patterns of population genetic characteristics that correlate with local endemicities and transmission intensity [21]. These results also demonstrate similar trends where parasites from Chulaimbo a mesoendemic region had the lowest genetic diversity compared to the Homa Bay and Kombewa *P*. *falciparum* strains.

There were no observed variations among populations, the only notable variations were among individuals in the population which is a product of other factors such as natural selection. This was confirmed by the negative non-significant Tajima’s *D* results that pointed to the existence of weak positive selection. Pfs47 may not only be selected against by the mosquito immune system which has been described to preferentially target D2 but also hosts antibodies [13, 34, 39]. The significant negative Fu’s *Fs* demonstrated that most of these observed rare alleles were in excess suggestive of a recent population expansion in the Western Kenya lowlands. In conclusion, there was no genetic differentiation among the three *P*. *falciparum* parasite populations. The excess of low frequency alleles may result from a population expansion or a positive selection. Significant negative values of Fu’s *Fs* are evidence for an excess of new haplotypes, a recent population expansion, or a selective sweep caused by genetic hitchhiking. Understanding interactions between circulating Pfs47 variants and mosquito immunity genes having implications on malaria transmission is crucial and should be considered when designing potential molecular targeted control interventions.

## Supporting information

S1 Table Primers and probes sequences used for the detection of malaria parasites. (DOCX)

S2 Table Distribution of Pfs47 haplotypes in western Kenya. (DOCX)
