# Supplementary material for: Genetic diversity and population structure of the human malaria parasite Plasmodium falciparum surface protein Pfs47 in isolates from the lowlands in Western Kenya
Source: PLoS One. 2021 Nov 29;16(11):e0260434. doi: 10.1371/journal.pone.0260434 (PMC8629314; doi:10.1371/journal.pone.0260434)
Supplement: S1 Table — (DOCX) [file pone.0260434.s001.docx]

**Genetic diversity and population structure of the human malaria parasite *Plasmodium falciparum* surface protein Pfs47 in isolates from the lowlands in Western Kenya**

Shirley A. Onyango^1,2^, Kevin O. Ochwedo^2,5^, Maxwell G Machani^3^, Collince J. Omondi^2,5^, Isaiah Debrah^2,7^, Sidney O. Ogolla, Ming-Chieh Lee^4^, Goufa Zhou^4^, Elizabeth Kokwaro^1^, James W. Kazura^8^, Yaw A. Afrane^6^, Andrew K. Githeko^3^, Daibin Zhong^4*^ and Guiyun Yan^4*^

**Supplementary Table 1: Primers and probes sequences used for the detection of malaria parasites**

| **Reagents** | **sequences** | **Other details** |
| --- | --- | --- |
| Pf-probes | 5'FAM-CATAACAGACGGGTAGTCAT-MGB3' | (FAM-MGB) |
| Pm-probes | 5'VIC-ATGAGTGTTTC'I'I'ITAGATAGC-MGB3' | (VIC-MGB) |
| Po-probes | 5'NED-CGAAAGGAA'I'I'ITCTTATT-MGB3' | (NED-MGB) |
| Pf forward primer | 5'ATTGCTI'ITGAGAGGTI'ITGTTACTTT3' |  |
| Pf reverse primer | 5'GCTGTAGTATTCAAACACAATGAACTCAA3' |  |
| Pm forward primer | 5'AGTTAAGGGAGTGAAGACGATCAGA3' |  |
| Pm reverse primer | 5'CAACCCAAAGACTITGATTTCTCATAA3' |  |
| Po forward primer | 5'AACCCAAAGACTTTGATTTCTCATAA3' |  |
| Po reverse primer | 5'CCGACTAGGTI'ITGGATGAAAGATI'I'IT3' |  |
