# Supplementary material for: Genetic diversity and population structure of the human malaria parasite Plasmodium falciparum surface protein Pfs47 in isolates from the lowlands in Western Kenya
Source: PLoS One. 2021 Nov 29;16(11):e0260434. doi: 10.1371/journal.pone.0260434 (PMC8629314; doi:10.1371/journal.pone.0260434)
Supplement: S2 Table — (DOCX) [file pone.0260434.s002.docx]

**Genetic diversity and population structure of the human malaria parasite *Plasmodium falciparum* surface protein Pfs47 in isolates from the lowlands in Western Kenya**

Shirley A. Onyango^1,2^, Kevin O. Ochwedo^2,5^, Maxwell G Machani^3^, Collince J. Omondi^2,5^, Isaiah Debrah^2,7^, Sidney O. Ogolla, Ming-Chieh Lee^4^, Goufa Zhou^4^, Elizabeth Kokwaro^1^, James W. Kazura^8^, Yaw A. Afrane^6^, Andrew K. Githeko^3^, Daibin Zhong^4*^ and Guiyun Yan^4*^

**Supplementary Table 2: Distribution of Pfs47 haplotypes in western Kenya**

| Haplotype | Codon changes | Number of samples per site | | | Total |
| --- | --- | --- | --- | --- | --- |
|  |  | Homa Bay | Chulaimbo | Kakamega |  |
| Hap_1 | E27D and P194H | 27 | 16 | 15 | 58 |
| Hap_2 | P194H and L240I | 8 | 2 | 2 | 12 |
| Hap_3 | P194H | 10 | 5 | 7 | 22 |
| Hap_4 | P194H and I304L | 3 | 0 | 1 | 4 |
| Hap_5 | E188D and P194H | 1 | 2 | 1 | 4 |
| Hap_6 | E27D, P194H and L240I | 5 | 2 | 1 | 8 |
| Hap_7 | P194H and N272I | 1 | 0 | 0 | 1 |
| Hap_8 | E27D, P194H and I248L | 3 | 0 | 1 | 4 |
| Hap_9 | E27D, E188D, P194H and I248L | 1 | 0 | 0 | 1 |
| Hap_10 | Same as 3D7* (No mutations) | 1 | 0 | 0 | 1 |
| Hap_11 | E27D, E188D and P194H | 2 | 2 | 2 | 6 |
| Hap_12 | E27D, P194H, I248L and N272I | 0 | 1 | 2 | 3 |
| Hap_13 | E27D, P194H and N272Y | 0 | 0 | 1 | 1 |

*NCBI Reference Sequence of 6-cysteine protein (*Plasmodium falciparum* 3D7): XP_001350182.1
